# Prospective observational study of surgery alone for locally advanced oral squamous cell carcinoma: a real-world study

**DOI:** 10.1186/s12903-024-03914-6

**Published:** 2024-01-31

**Authors:** Zhen-Hu Ren, Keyue Liu, Yiming Chen, Zhi-Min Yang, Kun Wu, Han-Jiang Wu

**Affiliations:** 1 Department of Oral and Maxillofacial surgery, Second Xiangya hospital of Central South University, No.139 Renmin Road, Changsha, 410011 Hunan China; 2 Department of Oral and Maxillofacial-Head and Neck Oncology, Shanghai Ninth People’s Hospital, Shanghai Jiao Tong University, School of Medicine, No.639, Zhizaoju Road, Shanghai, 200011 China

**Keywords:** Advanced oral squamous cell carcinoma, Surgery, Radiotherapy, Prognosis

## Abstract

**Introduction:**

A prospective observational study was modified to assess the efficacy of surgery alone for the treatment of locally advanced oral squamous cell carcinoma. (LA-OSCC)

**Materials and methods:**

This prospective, single-institution, single-arm study involved 174 patients who underwent major surgery for LA-OSCC. Participating patients did not receive postoperative radiation. After initial curative treatment, patients were routinely monitored via clinical examination and imaging. The follow-up period was 3–70 months. Tumour recurrence and death were considered as the Clinical End Point in Research.

**Results:**

The 5-year overall survival (OS), disease-free survival (DFS), and locoregional control rates for 174 patients were 66.7% (95% confidence interval [CI], 59.8 to 73.6), 66.1% (95% CI, 59.2 to 73.0), and 82.4% (95% CI, 76.5 to 88.3), respectively.

**Conclusion:**

A study of patients with LA-OSCC treated with surgery alone may have the optimal therapeutic impact for LA-OSCC, as evidenced by solid data for our next RCT trial. This conclusion still needs to be validated in higher-level RCTs.

Head and neck squamous cell carcinoma (HNSCC) originates in the oral cavity, oropharynx, larynx, or hypopharynx. It is the sixth most common cancer in terms of incidence worldwide, with over 600,000 cases identified annually. Forty to fifty% of HNSCC patients will survive for more than five years [1, 2]. Oral squamous cell carcinoma (OSCC) is the most common HNSCC. Multidisciplinary treatment, comprising surgery, radiation, and chemotherapy, is the main treatment approach for OSCC [3, 4]. With the advancement of surgery, radiation, chemotherapy, and biological treatment, the quality of life of patients with OSCC has substantially increased, but their prognosis has not improved, particularly for locally advanced OSCC (LA-OSCC) [5, 6], stage III-IVa and IVb according to the AJCC/UICC 7th edition.

Comprehensive treatment increases the number of problems and the social and financial burdens on patients. Numerous experienced clinical oncologists have discovered that the prognosis for many LA-OSCC patients is favourable even without postoperative adjuvant therapy such as radiation [7, 8]. Similarly, our team’s findings showed that patients with LA-OSCC did not require postoperative irradiation (PORT) to achieve a favourable prognosis, especially when [9] tongue cancer [10], buccal cancer and posterior oral cavity cancer are treated via high-quality surgical tumour excision [11]. For literature evaluation, most previous investigations were retrospective and thus the degree of evidence was low.

The objective of this prospective, observational study was to evaluate the efficacy of surgery alone for LA-OSCC. This offered substantial evidence for reevaluating whether postoperative radiation is needed for LA-OSCC.

## Materials and methods

### Patients and tumour characteristics

This single institution, single arm, prospective study was undertaken with 174 LA-OSCC patients who underwent radical surgery in the Department of Oral and Maxillofacial Surgery at the Second Xiangya Hospital of Central South University between February 2010 and April 2016. All patients with OSCC were surgically treated prospectively according to a protocol authorized by our institutional review board. The following inclusion criteria were used: (1) histologically verified OSCC; (2) cT3-4N0-3M0 or cT1-4N1-3M0; and (3) between 18 and 80 years of age. The exclusion criteria were as follows: (1) previously treated OSCC; (2) severe concurrent disease; (3) active multiple primary tumours; and (4) radiation received following surgery. Before initiating a procedure, we must tell both patients and their family members. In this study, the primary outcomes were overall survival (OS) and disease-free survival (DFS). The secondary outcomes included locoregional control, complications, quality of life and in-hospital cost. Almost all the patients had a history of smoking and alcohol consumption, and all the patients were negative for HPV infection.

### Treatment planning

Within one month before therapy, preoperative assessments comprised a physical examination, laryngoscopy, ultrasonography, imaging scan (CT and MRI), and 18-fluoro-odeoxyglucose PETCT (if necessary). All patients were advised of the protocol’s contents and needed to sign informed consent forms. Participating patients will not receive postoperative radiation. Our team was dedicated to clinical research on the surgical treatment of OSCC. A large number of clinical cases have been summarized through long-term clinical investigations. Surgical resection of OSCC, which we refer to as anatomic unit (subunit) resection, has been considerably improved over time [9, 12]. The main lesion was removed using anatomical unit resection surgery (AURS). The patient underwent radical excision of the main lesion and neck dissection (supraomohyoid with level I-III or radical and modified radical with level I-V) with suitable reconstruction (pedicle or free flap). A frozen pathology examination was conducted to confirm that the surgical margin was appropriate.

### Follow-up

Patients were instructed to return to the outpatient department every month (or even every two weeks for some high-risk patients) for the first 12 months, every three months for the second 12 months, every six months for the third 12 months, and then annually thereafter. Ultrasonography of the head and neck was conducted at each follow-up appointment, MRI or CT was performed every 3 to 6 months, PETCT was performed annually, and chest X-rays were obtained every six months if recurrence of the tumour was suspected.

### Statistical analysis

For statistical analysis, SPSS 23.0 (SPSS, Armonk, New York, United States) was utilized. The Kaplan–Meier technique was used to estimate locoregional control, DFS, and OS, and the log–rank test and the Cox proportional hazards model were used to compare them. *P* value < 0.05 was considered statistically significant.

## Results

There were 174 patients, 162 men and 12 women, ranging from age 22 to 76 years. The median duration of patient follow-up was 43.5 months.

The most prevalent tumour region was the tongue, followed by the buccal, mouth floor, and gingiva. Most of these tumours exhibited moderate differentiation. All patients were in stages III or IV, with two individuals in stage IVB and none in stage IVC (Table 1).

**Table 1:** Characteristics of the patient and the tumor

Characteristics	*n* = 174
**Age (midian) / years**	22–76(50)
**Sex**	
Male Female	162 12
**Location of primary tumor**	
Tongue Buccal Mouth floor Gingiva	99 46 18 11
**T stage**	
T1 T2 T3 T4	41 45 68 20
**N stage**	
N0 N1 N2 N3	51 59 63 1
**ECS**	
No lymph node involvement - +	51 73 50
**Pathology stage**	
III IVA IVB	87 85 2
**Pathologic margins of resection**	
Negative Positive	174 0
**lymphatic embolization**	
Negative Positive	124 50
**depth of invasion**	
≤ 0.5 cm 0.5 cm<doi ≤ 1 cm >1 cm	21 73 80

During the follow-up, 58 individuals were found to be deceased. At the end of the fifth year, the equivalent OS rates were 66.7% (CI: 59.8 to 73.6). The OS rates were 84.3% for N0, 89.4% for the N1, 42.6% for the N2, and 0% for N3 (Table 2, P0.001). The pathology stage accurately predicted the prognosis of patients (P0.001); the OS for stages III, IVA, and IVB were 83.7%, 58.2%, and 0%, respectively. OS was substantially linked with N stage, pathology stage, and extracapsular spread (ECS) (P0.001). OS was 75.1% without ECS and 49.6% with ECS. The relationship between tumour location (*P* = 0.770) and T stage (*P* = 0.307) and OS was not significant. Survival rates for T1, T2, T3, and T4 tumours were 70.5%, 59.4%, 76.5%, and 75.0%, respectively. Survival rates were as follows: 71.5% for tongue cancers (*n* = 99), 67.1% for buccal region tumours (*n* = 46), 66.7% for mouth floor tumours (*n* = 18), and 81.8% for gingival region tumours (*n* = 11). N stage (*P* = 0.001) and ECS (*P* = 0.024) were significantly related to OS in the multivariate analysis.

**Table 2:** Univariate evaluation of 5-year local control, disease-free survival, and overall survival

Characteristics	*n* = 174	Loco-regional control (%)	Disease-free survival (%)	Overall survival (%)
**Location of primary tumor**				
Tongue Buccal Mouth floor Gingiva *P*-value	99 46 18 11	88.6 69.6 71.3 100 *0.012*	71.6 67.1 61.1 81.8 *0.711*	71.5 67.1 66.7 81.8 *0.770*
**T stage**				
T1 T2 T3 T4 *P*-value	41 45 68 20	87.2 71.7 86.5 83.3 *0.262*	70.5 57.4 76.5 75.0 *0.281*	70.5 59.4 76.5 75.0 *0.307*
**N stage**				
N0 N1 N2 N3 *P*-value	51 59 63 1	90.1 93.2 64.0 0.0 *< 0.001*	84.3 89.4 41.1 0.0 *< 0.001*	84.3 89.4 42.6 0.0 *< 0.001*
**ECS**				
No lymph node involvement - + *P*-value	51 73 50	90.1 84.4 70.2 *0.062*	84.3 73.7 49.9 *< 0.001*	84.3 75.1 49.6 *< 0.001*
**Pathology stage**				
III IVA IVB *P*-value	87 85 2	89.6 76.4 0.0 *< 0.001*	83.7 57.6 0.0 *< 0.001*	83.7 58.7 0.0 *< 0.001*
**Depth of invasion**				
≤ 0.5 cm 0.5 cm<doi ≤ 1 cm >1 cm *P*-value	21 73 80	*80.9* *83.5* *82.5* *0.960*	*61.9* *50.7* *50.0* *0.607*	*61.9* *50.7* *48.8* *0.559*

During follow-up, 58 patients experienced recurrence. The DFS over five years was 66.1% (95% confidence interval [CI]: 59.2 to 73.0%). DFS was 84.3% for N0, 89.4% for the N1, 41.1% for the N2, and 0% for N3 (Table 2, *P* = 0.001). The pathology stage was also able to accurately predict the prognosis of patients (P0.001); the DFS rates for stages III, IVA, and IVB were 83.7%, 57.5%, and 0%, respectively. ECS was significantly linked to DFS (*P* < 0.001). DFS was 75.1% without ECS and 49.6% with ECS. The relationship between tumour location (*P* = 0.711) and T stage (*P* = 0.281) and DFS was not statistically significant. DFS was 71.6% for tongue tumour patients (*n* = 99), 67.1% for buccal tumour patients (*n* = 46), 61.0% for mouth floor tumour patients (*n* = 18), and 81.8% for gingival tumour patients (*n* = 11). The relative DFS rates for T1, T2, T3, and T4 cancers were 70.5%, 57.4%, 76.5%, and 75.0%, respectively. N stage (*P* = 0.002) and ECS (*P* = 0.024) were significant predictors of DFS according to the multivariate analysis.

The five-year rate of regional loco control was 82.4% (Tables 3 and 95% confidence interval [CI]: 76.5–88.3%). Intriguingly, in univariate analysis, only tumour site (*P* = 0.012), N stage (*P* < 0.001), ECS (*P* = 0.062), and clinical stage (*P* < 0.001) had an effect on the rate of locoregional control, whereas T grade (*P* = 0.262) did not. The multivariate analysis revealed that tumour site was significantly associated with regional control (*P* = 0.033).

**Table 3:** Multivariate analysis for local control, disease-free survival, and overall survival

Characteristics (*n* = 174)	Loco-regional control	disease-free survival	overall survival
Rate 95%CI	82.4% (76.5-88.3%)	66.1% (59.2-73.0%)	66.7% (59.8-73.6%)
*P*-value			
Location of primary tumor T stage N stage ECS Pathology stage Depth of invasion	0.033 0.725 0.122 0.345 0.447 0.830	0.744 0.507 0.002 0.027 0.229 0.256	0.828 *0.396* 0.001 0.024 0.204 0.125

Disease-free survival and overall survival rates decreased with increasing tumour invasion depth, but there was no significant difference. Of all the patients, only 50 reported lymphatic embolism, and there was almost no invasion of blood vessels and nerves. For other postoperative complications, there were 4 patients with venous crisis of the free flap and 3 patients with exposed titanium plate infection.

## Discussion

The major objective of this prospective study was to evaluate the efficacy of surgery-only treatment for LA-OSCC. To examine the requirement of PORT for the treatment of LA-OSCC and to provide data to support future randomized clinical trials (RCT). Under the assumption of correct surgical concept and sufficient radical operation, the optimal prognosis for patients with LA-OSCC can be obtained solely with surgery (5-year overall survival (OS) was 66.7%, disease-free survival (DFS) was 66.1%, and locoregional control rates were 82.4%).

In addition, the findings of this study revealed that tumour site, N stage and clinical stage, T grade, and ECS were substantially linked with prognosis. Multivariate analysis revealed that N stage (*P* = 0.001) and ECS (*P* = 0.024) were strongly associated with OS, N stage (*P* = 0.002) and ECS (*P* = 0.024) were significantly linked with DFS, and tumour location (*P* = 0.033) was significantly associated with locoregional control.

In our previous studies, for patients with advanced OSCC, surgical treatment was still the main mode of comprehensive treatment, even when combined therapy was used. The scope of surgical resection is significant to the overall prognosis of the patient. We developed a novel concept of the posterior oral anatomical complex (POAC) in past research, and the dissection of the muscle surrounding the tumour is the most important part of primary resection. Once the tumour invades the muscle, a phenomenon similar to graphite conduction occurs. The tumour may even metastasize far along the vertical axis of the muscle, while the metastasis along the horizontal axis will be slow, as if encountering a barrier.

This could be the first prospective trial to investigate the treatment of LA-OSCC with surgery alone. In Japan, a multicentre analysis of oral squamous cell carcinoma patients with single lymph node metastasis with ECS found that PORT was related to higher disease-specific survival (DSS) and overall survival (OS) rates than surgery alone [13, 14]. Wolff, a German researcher, found that PORT greatly improved locoregional control but had no effect on DFS in patients with LA-OSCC [15]. The 5-year survival rate was 54% in the PORT group compared to 71% in the surgery-alone group (*P* = 0.002), and a greater proportion of patients who received radiation developed locoregional recurrence than those treated by surgery alone [8]. Most of the evidence in the NCCN guidelines for HNSCC, particularly OSCC, is derived from retrospective research. Although surgery followed by radiotherapy is recommended for patients with local advanced cancer, a certain portion of patients do not receive postoperative radiotherapy for several reasons, among which the patients’ will, surgeons’ preference and tolerance to postoperative radiotherapy are still the main reasons. Few RCT studies have confirmed the prognostic advantage of PORT for OSCC patients. Similar to the findings of certain physicians’ studies, we observed in our clinical work that patients with OSCC did not benefit significantly from radiotherapy [16, 17] and that radiotherapy even resulted in several major complications [18, 19]. Early side effects of radiation therapy for head and neck squamous cell cancer include oropharyngeal mucosa (mucositis), lack of saliva (xerostomia) and more. Unfortunately, a series of late complications of radiotherapy, such as radiation caries, trismus, and osteoradionecrosis, were observed. Tissue necrosis is an important late complication of radiotherapy. Osteoradionecrosis (ORN) represents a particularly morbid late effect of radiation therapy for HNSCC that can significantly affect cosmetic and functional outcomes for patients [21, 22]. A previous retrospective study showed that the incidence of carotid artery stenosis increased over time in patients with head and neck squamous cell carcinoma who received radiotherapy after surgery [20, 23]. Radiation therapy also makes follow-up more difficult.

Our team is dedicated to researching the impact of PORT on the prognosis of LA-OSCC patients. The retrospective study data (unpublished) demonstrated that PORT does not increase the survival rate of LA-OSCC patients. The findings of this study indicate that LA-OSCC can be effectively treated with surgery alone. These findings show that PORT may not be needed in the sequential treatment of HNSCC [24]. Based on the prior study, our next research plan is to conduct an RCT on this topic. We intend to answer with a greater degree of evidence whether PORT is needed for the sequential treatment of LA-OSCC.

There are still some limitations to our study: it was a prospective observational study, and further RCTs are needed to verify the findings’ accuracy, which is our next step. In addition, some patients in our study were followed up through telephone interviews; therefore, the precise period of tumour recurrence in these individuals may not be accurate.

**Fig. 1:**
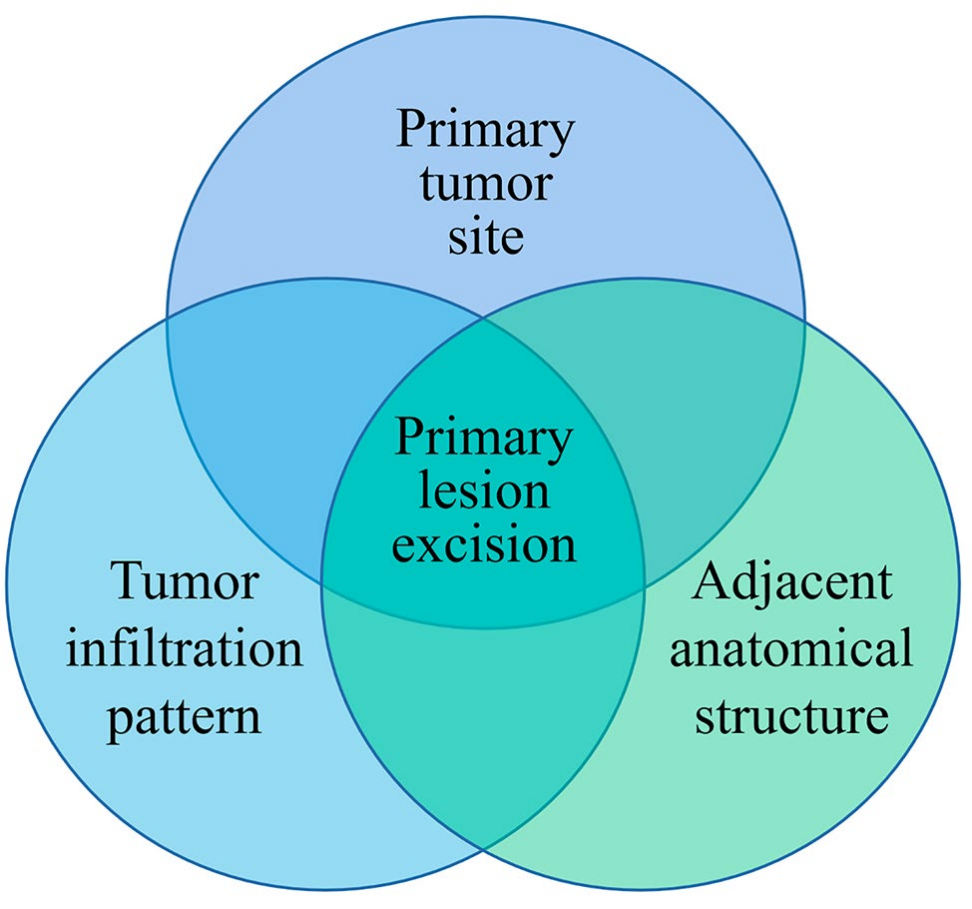
Three crucial parameters for the surgical treatment of LA-OSCC

The location, morphological and infiltrating characteristics of the tumour are three crucial factors that will affect the therapeutic response of surgical treatment for LA-OSCC (Fig. 1). The outcome of this study will provide robust data support for our subsequent RCT study. This is a study of the results of surgery alone versus radiation after surgery in patients with LA-OSCC.

## Conclusion

This prospective study suggests that surgical treatment alone may have a better therapeutic effect, and this conclusion still requires validation in higher-level RCTs.

## Data Availability

The datasets generated and analysed during the current study are not publicly available due the sensitive nature of questionnaire information for the study community but are available from the corresponding author on reasonable request.
